# Intraventricular infusion of clusterin ameliorated cognition and pathology in Tg6799 model of Alzheimer’s disease

**DOI:** 10.1186/s12868-018-0402-7

**Published:** 2018-01-25

**Authors:** Xue-Mei Qi, Cheng Wang, Xing-Kun Chu, Gen Li, Jian-Fang Ma

**Affiliations:** Department of Neurology and Institute of Neurology, Ruijin Hospital Affiliated to Shanghai Jiao Tong University School of Medicine, No. 197, Road Ruijin Second, Shanghai, 200025 People’s Republic of China

**Keywords:** Clusterin, Alzheimer’s disease, LRP-2, Amyloid

## Abstract

**Background:**

Alzheimer’s disease (AD) is characterized by the deposition of amyloid-β (Aβ) in brain parenchyma and cerebral blood vessels as cerebral amyloid angiopathy (CAA). Clusterin, a chaperon protein associated with Aβ aggregation, toxicity and transport through blood–brain barrier, may play a key role in the development of AD. Recently, clusterin peptide D-[113–122] was shown to mimic clusterin’s function and exerted therapeutic effect in atherosclerosis. In this study, we investigated whether this clusterin peptide also affected (Aβ) deposition in AD transgenic mouse.

**Results:**

Using a micropump, synthetic peptide 113–122 of clusterin protein (20 μg/200 μl) was infused into the lateral ventricle of 8-month 5 × FAD transgenic mouse model (Tg6799), for 2 weeks. Water-maze testing showed an improved cognitive function of the Tg6799 mice treated with clusterin. Immunocytochemistry and quantitative analysis revealed that intraventricular (icv) administration of clusterin peptide in Tg6799 mouse reduced Aβ plaques as well the severity of cerebral amyloid angiopathy. Enzyme-linked immunosorbent assay demonstrated a decreased in the soluble levels of Aβ (Aβ40 and Aβ42) in the brain. Western-blot revealed an increased level of LRP-2 after clusterin peptide treatment.

**Conclusion:**

These results suggest that icv infusion of clusterin peptide D-[113–122] offers a promising therapeutic approach to reduce Aβ deposition as well as CAA. The LRP2-mediated clearance system might be involved in the mechanism of these effects.

## Background

Alzheimer’s disease (AD) is a common neurodegenerative disease in elderly. Pathologically, it is characterized by abnormal amyloid deposition such as amyloid plaque and cerebral amyloid angiopathy (CAA), neurofibrillary tangle and neuron loss. Amyloid-beta (Aβ) is considered to play a key role in AD pathological processes [1]. Vascular clearance of Aβ was one of mechanisms contributing to the pathogenesis of AD [2–4] and new therapeutic method targeting on neurovascular system has made a crucial development for AD treatment [5, 6].

Clusterin, also named apolipoprotein J, is a major glycoprotein firstly identified in ram rete testis fluid [7] and recently shown to play an important role in AD [8]. Genetic polymorphisms of clusterin were found to be associated with risk of AD in different populations [9–14]. Vitro studies demonstrated that clusterin could bind amyloid β (Aβ) to prohibit aggregation by its molten globule domains [15]. In addition, clusterin was shown to bind low density lipoprotein receptor-2 (LRP-2) to facilitate Aβ clearance [16, 17]. This pathway could cooperate with Apolipoprotein E, another well-known risk factor of AD to maintain vascular clearance of Aβ [18, 19].

Interestingly, a clusterin peptide synthesized from D-amino acids corresponding to residues 113–122 was found to mimic the function of clusterin. Navab et al. [20] reported that clusterin peptide D-[113–122] inhibited high-density lipoprotein (LDL)-induced monocyte chemotactic activity and improved HDL inflammatory properties in apoE-null mouse. However, it remained unknown whether this peptide could also mimic the function of clusterin on Aβ metabolism. In addition, Takker et al. [21] found that intraventricular (icv) delivery of Aβ antibody was superior to systemic infusion for modulating Aβ deposition, especially for CAA. So in this study, we investigated whether intraventricular administrating clusterin peptide D-[113–122] could reduce amyloid deposition and hence improve memory impairment in an AD transgenic mouse.

## Methods

### Animals

All experiments were performed using female 5 × FAD mice (Tg6799 mice) at 8 months which harbor mutations in amyloid precursor protein (APP) and presenilins (PS1) and cause familial Alzheimer’s disease (FAD) [22]. In Tg6799 mice, significant amyloid deposition developed at 6–9 months and impaired memory at 4–5 months. For all experiment, mice were divided into three groups: no treatment (n = 10), saline treatment (n = 10) and clusterin peptide D-[113–122] treatment group (n = 10). All mice were performed according to the National Institutes of Health Guide for the Care and Use of Laboratory Animals with the approval (permit number: SYXK-2011-0113) of the Scientific Investigation Board of Shanghai Jiao Tong University School of Medicine, Shanghai, China. Protocols were performed with the approval of the Institutional Animal Care and Use Committee (IACUC) at Shanghai Jiao Tong University, Shanghai, China.

### Clusterin peptide D-[113–122] preparation

Clusterin protein D-[113–122] peptides were corresponding to LVGRQLEEFL residue of amino acid and synthesized by Sangon Blotech (Shanghai). Clusterin peptide D-[113–122] powder was dissolved in 1% DMSO solution and diluted in saline to a final concentration of 20 μg/200 μl. 200 μl clusterin peptide was infused into lateral ventricle of 8-months-old Tg6799 transgenic mouse by using micro pump for 2 weeks.

### Stereotactic administration

Mice aged 8 months were anesthetized with 5% chloral hydrate and then mounted in a stereotactic apparatus (David Kopf instrument) for intracerebroventricular injections. Injections of Clusterin polypeptide D-[113–122] (20 μg/200 μl) or saline were conducted using ALZET Brain Infusion Kit 3 and ALZET Mini-osmotic Pump Model 2002 (200 μl, number: 0008851). Briefly after making a middle skin incision in the cranial skin, the pump filled with solution was placed into a formed subcutaneous pocket on the back of the mouse. The brain infusion cannula of the Brain Infusion Kit was implanted into the lateral ventricle. Coordinates of the injection sites relative to bregma were anteroposterior (AP): − 0.1 mm, lateral (L): + 0.9 mm, dorsoventral (DV): − 2.5 mm. After sufficient awakening from anesthesia, animals were returned to their cages for 14 days. Then a small incision was made in the skin of mouse after anesthetized and the pump reservoir was removed. The brain infusion cannula was left in place. After surgery the animal was returned to the cage for 4 days. Then the mouse underwent cognitive testing.

### Morris water maze test

The Morris water maze is a classic behavioral test to evaluate spatial learning and memory function [23]. It is an open circular pool with 120 cm in diameter that is filled with 20 °C opaque water (dyed white with food colorants) located in a room with extra-maze cues. A high-resolution camera was suspended over the center of the pool and its images were monitored by a video-tracking system (Morris Water Maze Video Analysis System (DigBeh-MM), Shanghai Jiliang Software Technology Co. Ltd, Shanghai, China). The pool was divided into four equal quadrants: Northeast (NE), Southeast (SE), northwest (NW) and Southwest (SW). A platform (9 cm in diameter) is located in the middle of the NE quadrant. The Morris water maze test included 1-day of training making the mice familiarized to the device, 4 consecutive days of acquisition training with a hidden platform (1 cm below the water, the platform was remained in a fixed location for the entire test),and 1 day of probe trial. On the first day animals were familiarized to the device. For the following acquisition training, animals were given a series of training at a rate of 4 trails per day for 4 consecutive days with the platform hidden below the water. For each trial the mouse must find a hidden platform located in the NE quadrant within 60 s and was left on it for an additional 20 s. Mice not finding the platform within 60 s were placed on it or guided to it gently and left on the platform for 20 s. Each mouse received four training trials per day with an average inter-trial interval of 15 min. The daily order of entry into individual quadrants was randomized. The escape latency and distance to reach the platform were recorded by the video-tracking system. On the following day, the platform was removed and a 60 s probe trial was conducted. The spatial memory was evaluated by comparing the time spent in the NE quadrant to the time spent in the 3 other quadrants.

### Immunohistochemistry

One day after the end of behavioral tests, Mice were anesthetized and perfused with phosphate-buffered saline (PBS) by cardiac perfusion. The brains were immediately removed and bisected. One hemisphere was frozen in − 80 °C for further protein analysis. The other hemisphere was fixed in 4% paraformaldehyde for 24 h followed by gradient dehydration in 15, 20 and 30% sucrose and then embedded in optimum cutting temperature embedding compound (OCT) (Miles Diagnostics, Elkhart, IN, USA) in a coronal orientation. OCT-embedded tissues were cut at 13 μm thickness. The brain sections were blocked with 10% goat serum, and then incubated overnight with polyclonal Aβ42 antibody (SIGMA, Anti-Amyloid Peptide β, Cleavage Site 42 antibody produced in rabbit, 1:1000) or Aβ40 antibody (Cell Signaling Technology, β-Amyloid (1–40 Specific) (D8Q7I) Rabbit mAb, 12990S, 1:800) at 4 °C overnight. PBS was used in place of primary antibody as a negative control. After incubation with secondary antibody (Jackson Immuno Research, Peroxidase AffiniPure Goat Anti-Rabbit IgG (H + L) 111-035-003, 1:1000), immunoreactivity was detected with DAB, and sections were counterstained with hematoxylin. Images of three sections through both anatomic regions of interest (entorhinal cortex and region of the hippocampus) were captured from each animal, and a threshold optical density was obtained that discriminated staining from background. The percent area occupied by the plaques was determined stereologically in the hippocampus section immunostained with Aβ42 antibody. CAA burden, expressed as percent area occupied by vessels with CAA (immunostained with Aβ40 antibody) in temporal cortex.

### Enzyme-linked immunosorbent assay

Homogenates of hippocampus and the surrounding temporal tissue were prepared. Measurement of soluble Aβ peptides were quantified by specific ELISA kits (human Amyloid beta 40 ELISA Kit, ExCell Bio EH039; human Amyloid beta 42 ELISA Kit, ExCell Bio EH040). Assays were performed on 96-well plates according to the manufacturer’s recommendations and reproducibility of the assay for Aβ 40 and Aβ 42 were: Inter-Assay CV < 10%, Intra-Assay CV < 10%. Briefly, the sample was added into precoated plate and following incubated for 4 °C overnight, each well of precoated plate was washed with washing buffer. Then the biotin-conjugated antibody solution was added into the well and incubated for 120 min at 37 °C. After washing, streptavidin-HRP was added and then incubated for 60 min at 37 °CR. After washing, substrate solution was added and then incubated for 10–15 min at room temperature in the dark. Following adding stop solution, the resulting was assayed at 450 nm using Bio Tek Synergy 4.

### Western blot analysis

The temporal cortex and hippocampus of the brain were dissected and tissues were analyzed by Western blot as previously described [24]. Homogenates of brain tissue (hippocampus, and the surrounding temporal cortex) were prepared in RIPA buffer (50 mmol/l Tris HCl (pH 8.0), 150 mmol/l NaCl, 1% NP-40, 0.5% sodium deoxycholate, and 0.1% sodium dodecyl sulfate [SDS]) with protease inhibitor cocktail and PMSF (Beyotime). After 30 min incubation on ice, the homogenates were then centrifuged at 12,000*g* for 15 min at 4 °C, then the supernatants were collected and protein concentration was determined by the BCA assay (Thermo). Equal amounts of protein from the supernatant were separated using 7.5% sodium dodecyl sulfate gel, and proteins were transferred to a nitrocellulose membrane after electrophoresis. The LRP2 protein was probed with primary antibody (Anti-Lrp2/Megalin antibody [EPR5875] ab129198, 1:5000) at 4 °C overnight. After three washes with TBST, the membrane was incubated in horseradish peroxidase-conjugated secondary antibody (Jackson Immuno Research, Peroxidase AffiniPure Goat Anti-Rabbit IgG (H + L) 111-035-003, 1:10,000) for 1 h at room temperature. A monoclonal mouse antibody against β-actin was used to control protein loading and transfer efficiency. Membranes were incubated in Immobilon Western Chemiluminescent HRP Subtrate reagent (Millipore) and exposed to X-ray film (Kodak, Rochester, NY/USA) and analyzed using the NIH Image J software.

### Statistical analyses

Data are present as mean ± SEM. Multiple comparisons were evaluated by two-tailed ANOVA and Turkey test for comparison between two groups with SPSS 19.0 and p value < 0.05 was considered statistically significant. All experiments were performed three times.

## Results

### Icv-delivering clusterin peptide D-[113–122] improved memory dysfunction of Tg6799 transgenic mouse

**Fig. 1:**
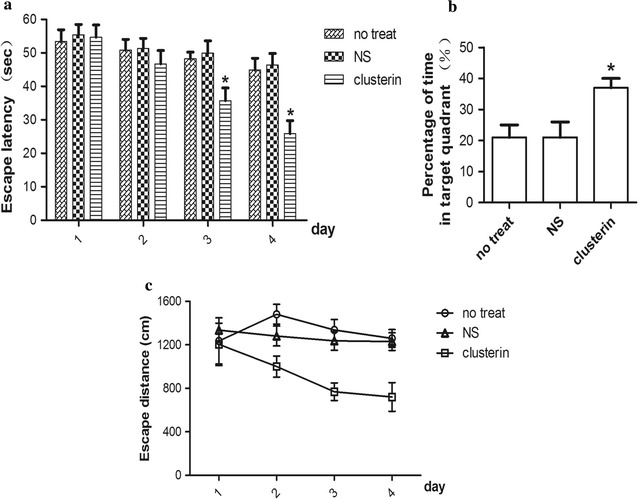
Tg6799 mice showed behavioral improvement after intraventricular (icv) infusion of clusterin peptide D-[113–122]. Tg6799 mice received nothing (non-treat), saline (NS) or clusterin peptide D-[113–122] via icv infusion for 2 weeks as indicated. Acquisition trial was performed four times a day for 4 consecutive days. Swimming time (**a**) and distance (**c**) to arrive at the platform was recorded. Non-treat and NS mice showed no improvement in swimming distance in comparison to clusterin treat group, and clusterin treat group exhibited less time-to-platform. On the following day a probe test was performed and clusterin treat group showed more time spent in the goal quadrant (**b**) as opposed to non-treat and NS mice. Data is presented as mean ± S.E.M. 8 months of age, n = 10 per group. *p < 0.05, one-way ANOVA followed by Turkey post hoc test

We firstly investigated whether clusterin peptide D-[113–122] infusion could improve the memory dysfunction of Tg6799 transgenic mouse by Morris water maze test. After intraventricular infusion of clusterin peptide for 2 weeks, we found that there was a significant difference of acquisition trial swimming time among three groups (F(2,27) = 4.54, p = 0.020 on day 3; F(2,27) = 5.57, p = 0.009 on day 4). The post Turkey test for comparison between two groups showed that the acquisition trial swimming time were significant decreased in clusterin group in comparison to non-treat group (p = 0.040 on day 3; p = 0.027 on day 4) and saline group (p = 0.035 on day 3; p = 0.015 on day 4) (Fig. 1a, c). The clusterin group also showed improvement of performance in swimming distance in 4 consecutive training trials (Fig. 1c). In the probe test the time spent in the goal quadrant was different among three groups (F(2,27) = 5.83, p = 0.008), and the following Turkey test showed that clusterin treated group showed more time spent in the goal quadrant (Fig. 1b) as opposed to non-treat (p = 0.020) and NS mice (p = 0.015). These results suggested that clusterin peptide D-[113–122] was able to improve memory impairment in AD transgenic mouse.

### Clusterin peptide D-[113–122] reduced Aβ deposition in Tg6799 transgenic mouse

**Fig. 2:**
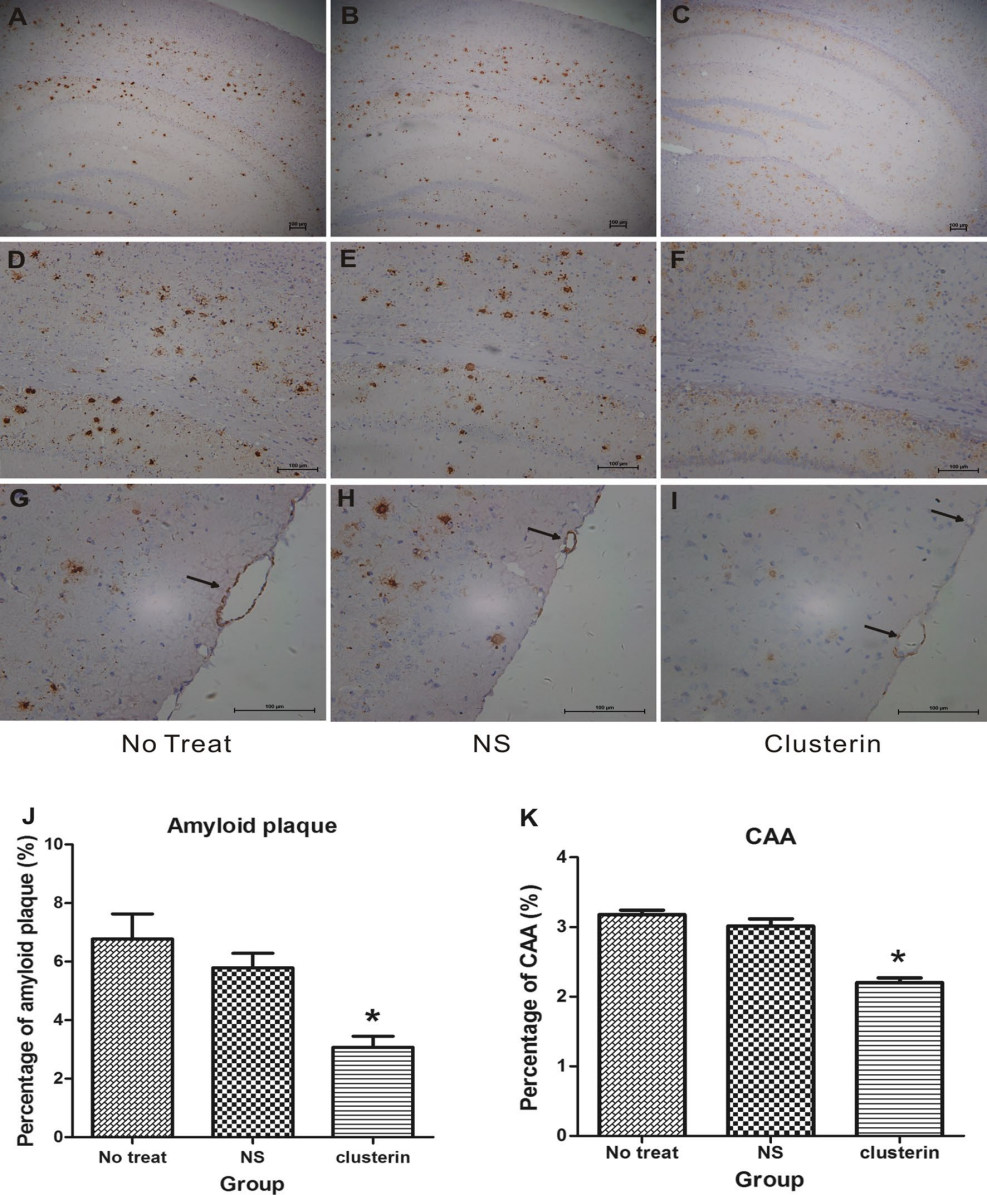
Decreased amyloid plaque and CAA following 2 weeks intraventricular (icv) treatment of clusterin peptide D-[113–122] in comparison to saline (NS) or nothing (non-treat). Images show amyloid plaque burden in hippocampus of Tg6799 mouse after icv-treatment of nothing (**A**, ** D**), saline (NS, **B**, **E**) and clusterin peptide D-[113–122] (**C**, **F**) as well as the CAA burden in cortical region of Tg6799 mouse after icv-treatment of nothing (**G**), saline (NS, **H**) and clusterin peptide D-[113–122] (**I**). Quantitative analysis of the percentage hippocampal area (**J**) occupied by amyloid plaque (Aβ42 immunohistochemistry study) and percentage cortical area (**K**) occupied by CAA (Aβ40 immunohistochemistry study) demonstrated a decrease after icv infusion of clusterin in Tg6799 mouse. Analysis involved 10 serial sections per mouse for cortex and 5 serial sections for hippocampus. 8 months of age, n = 5 per group. Data are presented as mean ± S.E.M. Scale bar = 100um. *p < 0.05; one-way ANOVA followed by Turkey post hoc test

We secondly examined whether Aβ deposition decreased in Tg6799 transgenic mouse after icv-administration of clusterin peptide D-[113–122]. In hippocampus slice, histoimmunochemistry by Aβ42 antibody showed that amyloid plaque load was different among three groups (F(2,12) = 15.14, p = 0.001), and the following Turkey test for comparison between two groups indicated that amyloid plaque was reduced in clusterin group (Fig. 2C, F) than non-treat group (p = 0.01) (Fig. 2A, D) and saline group (p = 0.04) (Fig. 2B, E). In temporal lobe cortical slice, histoimmunochemistry by Aβ40 antibody showed that there was a significant difference of CAA among three groups (F(2,12) = 27.79, p < 0.001) and the post Turkey test showed that CAA was reduced in clusterin group (Fig. 2I) than non-treat group (p < 0.001) (Fig. 2G) and saline group (p = 0.001) (Fig. 2H). These results suggested that the improvement of memory impairment after clusterin peptide D-[113–122] treatment might be mediated by reducing Aβ deposition in AD transgenic mice.

### Clusterin peptide D-[113–122] decreased soluble Aβ level in Tg6799 transgenic mouse

**Fig. 3:**
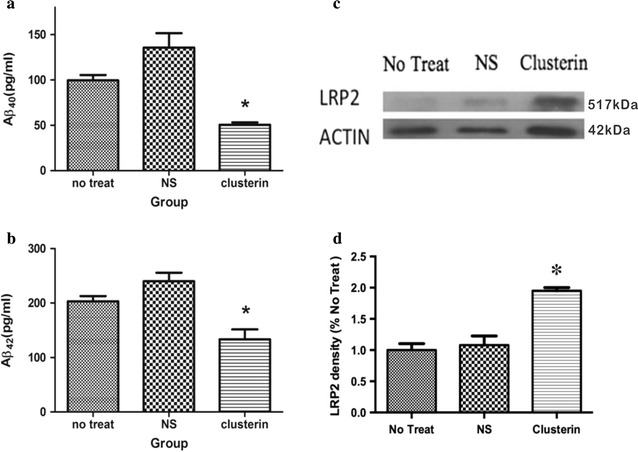
Soluble Aβ decreased and LRP-2 increased after intraventricular (icv)-delivering clusterin peptide D-[113–122]. Quantification of the soluble Aβ levels in hippocampus and the surrounding temporal cortex by ELISA revealed decreased soluble Aβ40 level (**a**) and soluble Aβ42 level (**b**). Western-Blot of protein lysates from hippocampus and the surrounding temporal cortex revealed increased LRP-2 expression in clusterin treated mice in comparison to non-treat and NS group (**c**). Density of LRP-2 was displayed in ratio to no-treat group (%) (**d**). 8 months of age, n = 9–10 per group. Data are presented as mean ± S.E.M. *p < 0.05; one-way ANOVA followed by Turkey post hoc test

In order to examine the possible mechanism of this therapeutic effect of clusterin peptide in reducing amyloid deposition, we used ELISA kit to measure the level of soluble Aβ42 and Aβ40 after clusterin peptide D-[113–122] treatment. Soluble Aβ42 were found different among three groups (F(2,25) = 7.76, p = 0.002) and the following Turkey test for comparison between two groups showed that soluble Aβ42 decreased in clusterin group than non-treat group (p = 0.028) and saline group (p = 0.002) (Fig. 3a). Soluble Aβ40 were also different among three groups (F(2,25) = 9.85, p = 0.001) and in the post Turkey test soluble Aβ40 were found decreased in clusterin group than non-treat group (p = 0.020) and saline group (p = 0.001) (Fig. 3b). The results showed that both soluble Aβ42 and Aβ40 decreased after clusterin peptide treatment.

### Clusterin peptide D-[113–122] increased LRP-2 level in Tg6799 transgenic mouse

Clusterin is also implicated in affecting Aβ clearance from brain and LRP-2 is the major receptor medicating the up-take of clusterin-Aβ complex [18, 25] so we further examined the expression of LRP-2 protein. We measured the level of LRP-2 protein after intraventricular infusion of clusterin peptide D-[113–122]. Using western-blot, we found that the expression of LRP-2 protein was found significantly different among three groups (F(2,25) = 41.90, p < 0.001), which increased in clusterin treating group when comparing to non-treat (p < 0.001) and saline group (p < 0.001) (Fig. 3c, d) in the post Turkey test, suggesting that LRP-2 protein was involved in the reduction of amyloid deposition after clusterin peptide treatment in AD transgenic mice.

## Discussion

In the present study, we investigated whether intraventricular injection of clusterin peptide D-[113–122] would affect AD and CAA pathology. Using Tg6799 transgenic mouse we found that intraventricular infusion clusterin peptide D-[113–122] for 2 weeks was able to ameliorate memory dysfunction of Tg6799 transgenic mouse, reduce amyloid plaque (mainly Aβ42) in hippocampus and CAA (mainly Aβ40) in cortical region of temporal lobe. In addition clusterin peptide decreased soluble Aβ level in the transgenic mouse. We further demonstrated that clusterin peptide treatment increased the expression of LRP-2.

Accumulating evidence has revealed that decreased clearance of Aβ plays a key role in AD pathogenesis, which suggests that promoting Aβ clearance could provide new therapeutic targets for AD treatment [26]. Recent years, many clinical trial targeting on anti-Aβ immunotherapies have failed in AD patients for its lack of efficiency or safety issues [27, 28], so new alternative therapeutic strategies are urgently needed. Clusterin, as a molecular chaperone, has been found to associate with AD, however it was still not fully understood how clusterin affect AD pathology. Plasma clusterin level was elevated early in AD patients and associated with severity of AD [29–31]. The role of plasma clusterin elevation remained controversial. Thambisetty et al. [32] found that a higher baseline plasma clusterin level was associated with slow brain atrophy rate, suggesting that elevated plasma clusterin was probably protective for neurodegeneration. Further studies have demonstrated that clusterin is involved in the toxicity, accumulation and clearance of Aβ in the brain [18, 33, 34]. Clusterin was also found to have high immunoreactivity in the arterioles and capillaries of AD and CAA patients, indicating clusterin is more likely to co-locate with Aβ40 rather than Aβ42, so that clusterin might mediate the elimination of Aβ40 through perivascular drainage pathway and be involved in CAA pathology [35]. In our study we found that intraventricular infusion clusterin peptide D-[113–122] was able to ameliorate memory dysfunction of Tg6799 transgenic mouse, reduce amyloid plaque (mainly Aβ42) in hippocampus and CAA (mainly Aβ40) in cortical region of temporal lobe. These results supported the protective role of clusterin and suggested that intraventricular infusion synthetic clusterin peptide might be a potential therapeutic method for modifying AD and CAA progress. And a recent research found a marked increase of CAA in the cerebrovasculature of APP-PS1 mice on a clusterin −/− background [36], which suggested that the perivascular drainage of Aβ was impaired in absence of clusterin. So clusterin may promote clearance of Aβ in the perivascular drainage route by preventing the deposition of Aβ on the cerebral vessels. However, knockdown clusterin protected against Aβ-induced apoptosis [37] and resulted in fewer fibrillar amyloid depositions [36, 38], indicating a harmful function of clusterin in amyloid deposition. Therefore, the effect of clusterin on Aβ aggregation and clearance is very complex and difficult to predict. These contradictory results demand further studies to reveal the pathological and physiological roles of clusterin in AD pathogenesis.

As we know, clusterin is one of extracellular chaperons that can bind targeted protein to prevent its aggregation [8, 15]. In vitro study, clusterin was found to sequestrate Aβ40 or Aβ42 oligomer to prevent its aggregation [39, 40], suggesting the neuroprotective effects of clusterin under physiological conditions. So if the therapeutic effect of clusterin was mediated by sequestrating Aβ40 or Aβ42 aggregates in Tg6799 transgenic mouse, we would expect increased soluble Aβ42 and Aβ40 after clusterin peptide treatment. However, this was not the case in our study which showed that soluble Aβ42 and Aβ40 were actually decreased in clusterin group, suggesting clusterin peptide might reduce the accumulation of Aβ42 and Aβ40 through other mechanism than its chaperone effect on aggregates of Aβ. Further studies are required to explore the effect of clusterin peptide on the biological behavior of brain amyloid beta as well as the mechanism involved in this process.

LRP family proteins play an important role in clearing Aβ42 and Aβ40 [41–43]. Drug such as simvastatin was shown to upregulate LRP to increase the Aβ clearance [41]. LRP-2/meglin, as a member of the LRP family protein, has been demonstrated to mediate the transport and cellular uptake of clusterin and Aβ-clusterin complex at the cerebrovascular endothelium and e choroid plexus epithelium [17]. Incubation of clusterin with Aβ40 could increase the LRP-2 mediated Aβ40 clearance in vitro study [16, 34]. In our present study, clusterin peptide treatment increased the expression of LRP-2, suggesting that the therapeutic effect of clusterin might be mediated through LRP-2 pathway. So it was possible that clusterin peptide increased the Aβ42 and Aβ40 clearance, decreased soluble Aβ42 and Aβ40 level, reduced the accumulation of Aβ42 and Aβ40 in plaque and CAA, and finally improved the memory impairment in Tg6799 transgenic mouse. However based on current data, it was not certain whether the elevation of LRP-2 expression was a direct change after clusterin peptide treatment or “by stander” effect. As we known, clusterin exhibits various function from chaperon, immune modulation and apoptosis [44]. For instance, clusterin could bind membrane attack complex (C5b-9) to modulate complement system [45], another important system involved in AD pathogenesis [46]. LRP had complement repeat-containing domains and was found to bind complement related protein [47, 48]. So it was also possible that clusterin peptide D-[113–122] induced complement activation first and then modulated LRP-2 expression afterwards.

In our present study, intraventricular injection was shown effective in improving memory dysfunction in AD animal model, however, this administration method is not applicable to patients. Moreover the effects of manipulating clusterin in AD transgenic mouse models or in vitro studies are difficult to predict [40, 49]. So before making the basic neuroscience finding into clinical effective therapies in patients, we need a better understanding of the detailed mechanisms of clusterin involved in the pathogenesis of AD.

## Conclusion

So far, there are no effective treatments to modify the progress of AD. Amyloid clearing therapies were demonstrated as an effective way to eliminate amyloid deposition in animal model [50–52]. Although clinical trials of these therapies were failed for different reasons [27, 28, 53], it remained a promising approach for AD modifying therapy. We found that intraventricular administration of clusterin peptide D-[113–122] was able to reduce amyloid accumulation and hence improve memory impairment in Tg6799 transgenic mouse through a mechanism involving LRP-2 mediated clearance system. These findings offer another interesting therapeutic approach for modulating AD progress. However, further studies are needed to reveal the exact mechanism how clusterin peptide D-[113–122] exert its therapeutic function and what role of LRP-2 plays in this treatment.

## Abbreviations

Aβ: amyloid β
CAA: cerebral amyloid angiopathy
AD: Alzheimer’s disease
LRP-2: low density lipoprotein receptor-2
icv: intraventricular
HDL: high-density lipoprotein
APP: amyloid precursor protein
PS1: presenilins1
FAD: familial Alzheimer’s disease
IACUC: Institutional Animal Care and Use Committee
PBS: phosphate buffered saline
ELISA: enzyme-linked immunosorbent assay
